# Comparison of Conventional Pressure-packed and Injection Molding Processing Methods for an Acrylic Resin Denture based on Microhardness, Surface Roughness, and Water Sorption

**DOI:** 10.1155/2022/7069507

**Published:** 2022-08-17

**Authors:** Elnaz Moslehifard, Tahereh Ghaffari, Hamidreza Abolghasemi, Solmaz Maleki Dizaj

**Affiliations:** ^1^Department of Prosthodontics, Faculty of Dentistry, Tabriz University of Medical Sciences, Tabriz, Iran; ^2^Department of Dental Biomaterials, Faculty of Dentistry, Tabriz University of Medical Sciences, Tabriz, Iran; ^3^Dental and Periodontal Research Center, Tabriz University of Medical Sciences, Tabriz, Iran

## Abstract

Polymethyl methacrylate (PMMA) is a widely used material in prosthetics and is used to fabricate denture bases. The main disadvantage of this material is its polymerization shrinkage which causes clinical problems during use. The present study aimed to investigate and compare the microhardness, surface roughness, and water sorption of a commercial acrylic resin denture, which were processed by two different methods including conventional and pressure-packed injection molding techniques. A total of 60 polymethyl methacrylate samples were prepared in two groups: conventional acrylic resin (vertex) for the compression molding method and injection acrylic resin (vertex) for the injection molding method (10 samples of each material per test). The microhardness test was performed using a Vickers microhardness test device, the surface roughness test was performed by using a profilometer, and the water sorption test was performed using a digital scale. Data were analyzed using an independent sample *t*-test with Statistical Package for the Social Sciences (SPSS), version 17. The significant level was considered to be 0.05. According to the results, there was a significant difference between microhardness, surface roughness, and water sorption of the samples in the two groups. The results of the independent *t*-test showed that the microhardness of injection vertex acrylic resin samples was significantly higher than that of conventional pressure-packed vertex acrylic resin samples (*P* value<0.05). Also, the surface roughness and water sorption of injection vertex acrylic resin samples were significantly lower than those of conventional pressure-packed vertex acrylic resin samples (*P* value <0.05). According to the obtained results, denture fabrication by the injection molding method can improve the quality and durability of dentures due to the increased microhardness, the decreased surface roughness, and the decreased water absorption of the denture base compared with the conventional method.

## 1. Introduction

Dentures have been used as a style of handling for replacing missing teeth since 700 B.C [1]. Polymethyl methacrylate (PMMA) is usually applied for prosthetic dental requests, including the production of artificial teeth, denture bases, dentures, obturators, orthodontic retainers, temporary or provisional crowns, and the repair of dental prostheses [2]. PMAMA is an appropriate and common biomaterial for dental applications due to its outstanding possessions including low density, *c*, low cost, and the simple manipulation of physic-mechanical possessions [2]. PMMA has been a widely used material in prosthetics and has been used to fabricate denture bases since 1937 [3]. Methacrylates are available in two types, self-cure and heat-cure [4].

Techniques for denture processing have the main impact on denture properties [5, 6]. Compression molding is a common method of acrylic resin curing [7]. The relative ease of curing acrylic resin with this method, the mastery of dentists and technicians, and no need for a special and expensive device are its advantages, but dimensional changes in the denture base during polymerization of the resin are among the disadvantages of this method [8]. A relatively new injection method has been developed to solve problems caused by the shrinkage of heat-cured acrylics. It is reported that these new materials and methods have improved many necessary properties such as flexural strength, transparency, flexibility, less water absorption, less residual monomer, fewer pores, and thus more dimensional stability. The new materials have no metal compounds and have a microcrystalline structure that ultimately makes the finishing and polishing much easier [4, 9].

Hardness is an important property of materials that indicates the resistance of materials to plastic deformation due to abrasion forces [10]. This feature is used to describe the abrasion resistance of materials, and lower hardness is usually associated with low abrasion resistance and susceptibility to scratches [11]. The low hardness value of acrylic denture bases indicates that dentures can easily be abraded and can make microcracks, so weakening the denture base leads to bacterial gathering [5]. The denture base hardness test evaluates the effect of denture cleaners, temperature changes, toothbrush, and toothpaste abrasion, as well as evaluates the effect of different polymerization systems on the surface properties of acrylic resins [12, 13]. Surface roughness is another property that affects the surface properties of denture bases. The surface roughness of dental materials may cause small traumas in the tissue and increase the entrapment of microorganisms that directly and indirectly play roles in increasing tissue damage and the incidence of oral diseases [14, 15]. Roughness can be considered a set of small irregularities and contradictions that are present on surfaces and affect wettability, bonding quality, and brightness [16]. Prosthetic appliances should have a smooth surface to maintain the health of oral tissues and reduce the entrapment of microorganisms and plaque accumulation [17]. Water sorption is another feature of materials to fabricate dentures. This feature has the capacity of plastic or polymers to absorb moisture from the environment. Water sorption can lead to denture base discoloration, halitosis, bad breath, dimensional instability of the denture, conduction of internal stresses, and susceptibility to cracks or denture failure [18, 19]. In other words, high water sorption affects the properties of materials and causes loss of mechanical properties such as fatigue limit, transverse strength, and hardness. Furthermore, water sorption causes three-dimensional expansion and thus can affect the dimensional stability of acrylic resins [20, 21] and reduce the lifespan of dentures in the oral cavity.

Due to the lack of information about injection techniques and their effects on the physical and mechanical properties of acrylics, the present study aimed to compare the microhardness, surface roughness, and water absorption of acrylic resins (vertex brand) prepared by compression and injection molding. The hypothesis of our work was performed to understand whether the processing technique, i.e., pressure-packed and injection molding, of vertex acrylic bases changes the micro-hardness, surface roughness, and water sorption.

## 2. Materials and Methods

### 2.1. Sample Preparation

**Figure 1:**
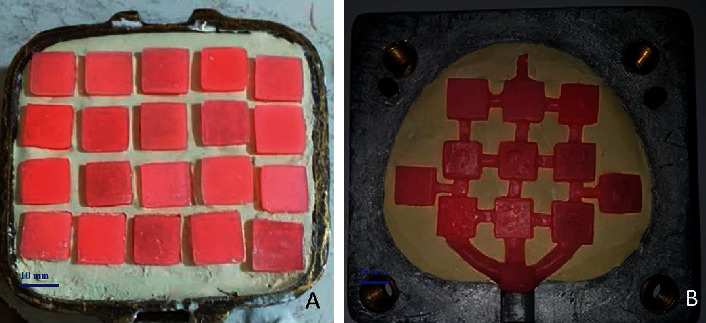
(a) Flasking of wax parts in the compression molding method, (b) Flasking of wax parts in the injection molding method.

Sixty samples were selected including cubic acrylics with dimensions of 12 × 12 × 3 mm (18, 19), prepared by two methods of compression and injection molding. To prepare the samples, 60 wax samples were fabricated (Tenatex Red Modeling Wax, Associated Dental Products Limited, Purton, Swindon, Wiltshire, SN5 4HT, UK) with dimensions of 12 × 12 × 3 mm, and the flasking steps were performed separately for each sample (Figure 1).

To prepare conventional heat-cured samples, wax samples were flasked after being impregnated with microfilm (Dandiran, Tehran, Iran). After the wax removal, powder and liquid of vertex (Vertex Dental, Zeist, Netherlands) were mixed according to the manufacturer's instructions in a ratio of 35 gr/14 ml and pressed flasks at 2 bar pressure. The flasks were then placed in a thermostat-controlled water bath at 70°C for 30 min and immersed at 100°C for 90 min and then in water to reach a degree of 40°C, and then, the samples were deflasked.

The preparation of injection samples began with the placement of wax samples in special flasks. After removing the wax, the vertex liquid and powder (Vertex Castavaria, Vertex Dental, Zeist, Netherlands) were mixed with a standard ratio of 1.7 g to 0.95 g, and then, it was injected into the flask. After 5 minutes, the flasks were put into a pressure pot at 2.5 bar and 55°C for 30 minutes, and then, the flasks were removed; the samples were removed from the flasks. Therefore, 10 samples were prepared from each material. Dimensions of samples were measured using a digital caliper (Mitutoyo Corporation, Japan). The surfaces of the samples were polished with aluminum oxide sandpapers with a hardness of #200, #400, and #800, respectively (made by STARCKE in Germany Matador, Grit Brand), ten times for each sample. Samples were polished by a person, and a new paper was used for each sample. After each polishing step, the samples were washed in an ultrasonic bath. Twenty samples (10 samples of each material) for each test were fabricated.

### 2.2. Microhardness Measurement

Vickers microhardness measurement of acrylic samples using a microhardness tester (made by SCTMC Company, Model HV-1000Z) in the Central Laboratory of Tabriz University was performed under a force of 30 grams at 30 seconds according to ISO 20759–1: 2013 [22]. Each sample was subjected to a hardness test 3 times (once in the center and twice in the sides), and we reported an average of 3 tests.

### 2.3. Surface Roughness

The surface roughness of the samples was measured using the profilometer device (SHARIF SOLAR Company) with a resolution of 0.01 *μ*m. After calibrating the device with a 0.8 mm long sample, we tested the prepared samples based on ISO/TR 14569–1:2007 [23].

### 2.4. Water Sorption

All samples were first dried to measure water sorption according to specification no. 1567–1981(ISO: 6887–1986) [24] for denture base polymers. To this end, the samples were transferred into an incubator at 37°C. Samples were repeatedly weighed at 24-hour intervals to reach a constant weight (the difference of each 24 hours should be less than 0.02 mg). They were then immediately weighed using a digital scale (model AS 310/C/2 made by RADWAG Company). Samples were immersed in distilled water at 37°C in a water bath for 30 days using a digital incubator (Behdad, Tehran, Iran). They were weighted again after this period using a digital scale (Kia Electronic Aras Co., Ltd., Tehran, Iran).

The following formula was used to calculate the amount of water sorption:

(1) $$\begin{array}{c} \text{water sorption}=\frac{m_{2}-m_{1}}{v}, \end{array}$$

where *m*_1_ is the sample weight (in micrograms) before immersion, *m*_2_ is the sample weight (in micrograms) after water immersion, and *v* is the sample volume (in cubic millimeters). We measured the dimensions of the samples using a digital caliper and used these dimensions to calculate the sample volume. We then measured water absorption in both groups for 30 days.

### 2.5. Statistical Analysis

The results were reported as descriptive statistical indices. The independent *t*-test was used to compare the variables between the two groups and utilized Statistical Package for the Social Sciences (SPSS), version 17 to analyze the data. A probability value of less than 0.05 was considered the significance level.

## 3. Results

After examining the data normality in the groups by the Kolmogorov–Smirnov test and comparison between the two groups, the independent *t*-test at a significance level of 0.05 was used.

The results of Table 1 indicated that the microhardness of acrylic bases of the injection vertex with a mean of 25.18 ± 4.30 was significantly higher than that of the conventional heat-cured vertex with a mean of 11.86 ± 1.28 (*P* value <0.001). The amount of surface roughness of acrylic vertex injection bases with a mean of 4.90 ± 1.12.12 was significantly less than that of the conventional heat-cured vertex with a mean of 7.58 ± 2.69 (*P* value 0.014) (Table 2), and the amount of water sorption of acrylic bases of the injection vertex with a mean of 21.29 ± 2.68 was significantly less than that of the conventional heat-cured vertex with a mean of 25.00 ± 2.54 (*P* value 0.005). These results are shown in Tables 2 and 3.

## 4. Discussion

According to the null hypothesis, the microhardness, surface roughness, and water sorption of vertex acrylic bases processed by two pressure-packed and injection molding methods were not different, but the null hypothesis was rejected as this present study indicates that there was a significant difference between the microhardness, surface roughness, and water sorption of the samples in the two groups. The results indicated that the microhardness of the injection vertex samples was higher than that of the common baking samples, and also, the surface roughness of the injection molding group was less than the samples of the compression molding group. Water sorption of injected molded acrylic resins was less than that of common acrylic resins.

The high value of surface microhardness of acrylic resin shows its high abrasion resistance, which impacts the durability and fracture resistance of a denture [5]. The polishing method can impact surface roughness [1]. The polishing method used in this study may affect the surface roughness and microhardness.

Song et al. conducted a study to investigate the hardness of injection thermoplastic denture bases. In the study, 6 types of available materials were selected from four groups of denture materials, including polyamide, polyester, acrylic resin, and polypropylene. Acritone (from the acrylic resin group) was harder than the other materials [25].

Our results are consistent with the study of Porwal et al. (2016) who examined the effects of denture cleaners on color stability, surface roughness, and denture hardness of different bases. Their results indicated that due to the use of denture cleaners, the hardness of heat-cured acrylic resin significantly decreased more than injection acrylic resin. The surface roughness of heat-cured acrylic increased more than acrylic injection [26]. However, in another report by Bahrani et al., the hardness and surface roughness of two types of acrylics polymerized with compression and injection molding methods were compared. They concluded that their surface hardness and roughness were not significantly different, which was inconsistent with the results of the present study. FuturaGen and Meliodent were used in the study, and the difference in results could be due to the difference in the type of acrylic [27]. Richmond et al. examined scratch resistance and surface roughness of two types of injectable polymethyl methacrylate (SR-Ivocap plus, Ipsyl 60 RV) and a type of resin prepared by the compression molding method (Trevalon). Their results indicated that SR-Ivocap plus showed the lowest roughness among the materials, and the results were consistent with those of the present study [13]. Abuzar et al. compared the surface roughness of polyamide (prepared by injection) and polymethyl methacrylate (prepared by compression molding) and concluded that the surface roughness of polyamide was higher than that of polymethyl methacrylate. Since the types of materials in the study were not similar to those of the present study, the results were not directly comparable with our results [14]. In a study by Kuhar and Funduk, there was not any significant difference between the roughness of heat-cured and injection acrylic resin. In the study, ProBase was selected as a heat-cured resin, and SR-Ivocap plus was selected as an injection acrylic resin. The difference between the results of the study and the present study might be due to the difference in brands of acrylic resins [28].

Generally, stabilizing the structure decreases water sorption and then enhances the flexural properties [6, 29]. The occurrence of reduced water sorption may also be due to the increased hardness [5].

The water sorption findings in the present study were similar to the findings of Pfeiffer and Rosenbauer who found that water sorption of the thermoplastic group (injection molding method) was significantly lower than that of the control group and polymethyl methacrylate (compression molding method) [30]. Jang et al. found that thermoplastic acrylic resin had less water sorption than conventional heat-cured resin, and their result was consistent with the present study [31].

The results of a study by Hemmati et al. were similar to those of the present study. They compared the water sorption of a thermoplastic PMAM and a conventional heat-cured acrylic, and the results indicated that the water sorption of thermoplastic PMAM was lower than that of the thermoset acrylic [32].

Ghasemi et al. compared water sorption of two types of injection acrylic resin (Ivo Base and Vertex) and a type of conventional acrylic resin (Meliodent). Ivo Base water sorption was lower than the other two substances, and Vertex had the highest water sorption. The finding indicated that, in addition to the molding method, the acrylic resin brand could also affect resin properties [33].

The conversion of a monomer to a complete polymer does not occur, and a variable amount of free or unreacted monomer remains in the polymerized resin during the polymerization reaction. The remaining monomer acts as a plasticizer and affects the mechanical properties of the acrylic resin [34].

Fabricating dentures by injection molding improves many necessary properties, including less water absorption, less residual monomer, fewer pores, and greater dimensional stability. These materials also have a microcrystalline structure [4, 9].

The acrylic resin molding method is effective on shrinkage due to polymerization and the amount of residual monomer. In the injection method, more complete polymerization is performed and the amount of the monomer remaining is less than in the conventional method. The dimensional stability of the material is also higher in this method. The amounts of the residual monomer and polymerization shrinkage affect the physical properties of acrylic resins. Therefore, microhardness, surface roughness, and water sorption of acrylic bases are affected by the amount of the residual monomer, and its plasticizer properties; hence, it seems that more microhardness and surface roughness, and less water sorption of injection-molded samples are due to greater dimensional stability and less residual monomer [24, 33].

These findings indicate better properties of acrylic resin polymerized by the injection molding method than conventional polymerized acrylic resin or compression molding method, and they are consistent with the results of the present study.

### 4.1. The Limitation of Study

The other properties such as flexural strength and fracture toughness should be evaluated for the prepared samples in this study. Besides, the other types of denture-based materials should be tested to validate the results of this study.

## 5. Conclusion

Based on the results of the current study, denture fabrication by the injection molding technique can progress the quality and durability of dentures owing to the enhanced microhardness, reduced surface roughness, and decreased water absorption of the denture base compared with the conventional process.

## Figures and Tables

**Table 1:** Microhardness in two types of acrylic resins in the study.

Type of acrylic resin	Minimum	Maximum	95% confidence interval		Mean	Standard deviation	*P* value
			Lower bound	Upper bound			
Injection vertex	20.10	29.41	20.88	29.38	25.18	4.30	<0.001
Conventional heat-cured vertex	10.16	13.88	10.58	13.14	11.86	1.28	

**Table 2:** Surface roughness in two types of acrylic resin in the study.

Type of acrylic resin	Minimum	Maximum	95% confidence interval		Mean	Standard deviation	*P* value
			Lower bound	Upper bound			
Injection vertex	3.5	5.9	3.7	6	4.9	1.12	0.014
Conventional heat-cured vertex	4.5	10.25	4.9	10.32	7.58	2.69	

**Table 3:** Water absorption in two types of acrylic resin in the study.

Type of acrylic resin	Minimum	Maximum	95% confidence interval		Mean	Standard deviation	*P* Value
			Lower bound	Upper bound			
Injection vertex	18.05	24.5	18.5	23.99	21.29	2.68	0.005
Conventional heat-cured vertex	22.45	28.62	22.47	27.58	25	2.54	

## Data Availability

The raw/processed data required to reproduce these findings can be shared after publication upon request from the corresponding author.
